# Using predicate and provenance information from a knowledge graph for drug efficacy screening

**DOI:** 10.1186/s13326-018-0189-6

**Published:** 2018-09-06

**Authors:** Wytze J. Vlietstra, Rein Vos, Anneke M. Sijbers, Erik M. van Mulligen, Jan A. Kors

**Affiliations:** 1 Department of Medical Informatics, Erasmus University Medical Centre, Rotterdam, 3015 GE the Netherlands; 2 Department of Methodology and Statistics, Maastricht University, Maastricht, 6200 MD the Netherlands; 3 Centre for Molecular and Biomolecular Informatics, Radboudumc, Nijmegen, 6525 GA the Netherlands

**Keywords:** Predicate, Provenance, Drug efficacy screening, Machine learning, Knowledge graph, Drug repurposing, Systems pharmacology, Computational pharmacology

## Abstract

**Background:**

Biomedical knowledge graphs have become important tools to computationally analyse the comprehensive body of biomedical knowledge. They represent knowledge as subject-predicate-object triples, in which the predicate indicates the relationship between subject and object. A triple can also contain provenance information, which consists of references to the sources of the triple (e.g. scientific publications or database entries). Knowledge graphs have been used to classify drug-disease pairs for drug efficacy screening, but existing computational methods have often ignored predicate and provenance information. Using this information, we aimed to develop a supervised machine learning classifier and determine the added value of predicate and provenance information for drug efficacy screening. To ensure the biological plausibility of our method we performed our research on the protein level, where drugs are represented by their drug target proteins, and diseases by their disease proteins.

**Results:**

Using random forests with repeated 10-fold cross-validation, our method achieved an area under the ROC curve (AUC) of 78.1% and 74.3% for two reference sets. We benchmarked against a state-of-the-art knowledge-graph technique that does not use predicate and provenance information, obtaining AUCs of 65.6% and 64.6%, respectively. Classifiers that only used predicate information performed superior to classifiers that only used provenance information, but using both performed best.

**Conclusion:**

We conclude that both predicate and provenance information provide added value for drug efficacy screening.

**Electronic supplementary material:**

The online version of this article (10.1186/s13326-018-0189-6) contains supplementary material, which is available to authorized users.

## Background

Knowledge graphs describe biomedical entities, such as diseases, proteins, or drugs, and their relationships [1]. They represent knowledge by subject-predicate-object triples, in which the predicate indicates the relationship between an entity pair (subject and object) [2]. A triple can also be enriched with provenance information, which consists of references to the sources where the triple is described. For example, the triple OPRM1-forms protein complex with-GNAT2 is described in the Reactome database [3]. Using triples, knowledge contained in a variety of sources, ranging from scientific articles to biomedical databases, can be incorporated within knowledge graphs [4].

Knowledge graphs have been applied to multiple problems in biomedical research, such as the extraction of disease biomarkers [5], identification of disease mechanisms [6], and numerous pharmacological use cases in the Open PHACTS project [7]. One of the most important use cases in computational pharmacology is the prediction of the health benefits of a drug over a placebo, i.e. its efficacy [8].

Many knowledge-graph methods have already been developed for predicting the efficacy of drugs [9–16]. Most of these methods do not use predicate or provenance information, but use the similarity between drugs to predict their efficacy for diseases. These methods count the number of common entities in a graph between two drugs, or between a drug and a disease. The underlying assumption of these methods is that a high number of common entities indicates similarity between drugs, which are therefore likely to be efficacious for the same diseases. They typically perform well for existing, well-characterized drugs. However, Guney demonstrated that the performance of similarity-based methods drops drastically when predicting the efficacy of drugs that are new, poorly characterized, or dissimilar to other drugs [17]. He considered the limited insight that these methods offer into the mechanisms behind the efficacy of the drugs as another drawback.

In other work, Guney et al. [13] determined the efficacy of drugs by measuring the distance in the graph between drug target proteins and disease proteins (i.e. the proteins coded for by the genes that are associated with a disease). The underlying assumption was that a shorter distance between drug targets and disease proteins was associated with a higher likelihood of efficacy. Although they described a coherent and plausible mechanism by which the efficacy of drugs could be explained, the performance of their method in determining the efficacy of drugs was moderate, with an area under the ROC curve of 66%.

Recent methods have included predicate information in knowledge-graph analyses [14–16]. Alshahrani et al. first transformed individual entities and predicates in their knowledge graph to numeric vector representations with the RDF2vec tool [14]. Machine learning was used on these vectors to predict new relationships for different pharmacological use cases. However, due to the complexity of the transformation performed by the RDF2vec tool, this method provides no insight into a possible functional mechanism. Weissenborn et al. created a knowledge graph based on a very large number of predicate types extracted from the biomedical literature, to which they applied machine learning [15]. They exclusively focused on literature and did not utilize the large amounts of knowledge contained in databases. Himmelstein et al. extracted paths of varying lengths between drugs and diseases from their knowledge graph [16]. Only a limited number of predicate types could be found in these paths; the majority of the entity types in their knowledge graph could only be connected to each other by a single predicate type. The predicate and entity types in these paths were combined to create so-called metapaths (e.g. “Compound–binds–Gene–associates–Disease”). Machine learning was used on all metapaths between a drug and a disease to classify whether it could be used as a treatment. Their decision to only include a limited number of predicates in their knowledge graph may under-utilize the information available in knowledge sources, which often describe multiple predicate types between the same types of entities. None of the methods mentioned above used provenance information.

Here, we investigate the added value of predicate and provenance information for drug efficacy screening by using them as features for a machine learning algorithm. We extract the predicate and provenance information from a comprehensive, commercially available knowledge graph, which contains knowledge from scientific literature as well as from a large number of databases. We compare our method with the state-of-the-art method of Guney et al., which does not use predicate and provenance information [13].

## Methods

### Knowledge graph

We used the Euretos Knowledge Platform (EKP), a commercially available knowledge graph [18]. The EKP is a generic platform, which contains information from 176 existing knowledge sources from a wide variety of domains in the life sciences. Biomedical entities such as proteins, drugs, or diseases are represented in the knowledge graph as vertices, each of which has one or more identifiers associated with it from external databases. The predicates and provenance between a subject and an object form a set of triples that define the edge between the vertices representing the subject and object. A path between two vertices is defined as a sequence of triples, or possibly a single triple, connecting the vertices.

Mappings between the entities in the different knowledge sources underlying the knowledge graph were made by matching their identifiers. The predicates in the knowledge sources were matched to a set of 203 predicate types, which are based on the predicates defined in the UMLS, extended with predicates from other knowledge sources [19]. If an exact match was not available, the predicates were manually mapped. If there were no explicit predicates in a database that was used as a knowledge source, the predicates were derived from the database schema.

To investigate the functional mechanisms behind the efficacy of drugs, we worked on the human protein level, similar to Guney et al. [13]. Knowledge graphs consisting solely of protein-protein interactions have been extensively used to investigate (interactions between) diseases, although these analyses typically do not use predicate or provenance information [20–22]. Sets of drug target proteins (henceforth referred to as drug targets) were used to represent one or more drugs, while sets of disease proteins were used as representatives of one or more diseases. A set consisted of one or more proteins. Apart from protein information, no information about the other entity types available in the knowledge graph was used.

**Fig. 1:**
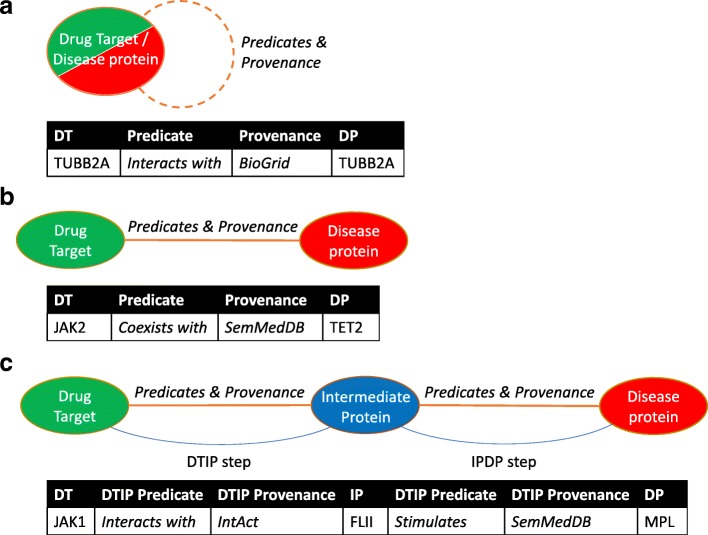
The three included relationship scenarios. The three scenarios of relationships between drug targets and disease proteins are shown along with examples which can be found in the knowledge graph. **a** Drug target (DT) and disease protein (DP) are the same protein. The protein may have a relationship with itself (dotted line). **b** DT and DP have a direct relationship. **c** DT and DP have an indirect relationship through an intermediate protein (IP). Indirect relationships consist of two steps (DTIP and IPDP)

When extracting the paths between drug targets and disease proteins from the EKP we distinguished three scenarios, as shown in Fig. 1. In the first scenario, a disease protein is also a drug target. Sometimes, these proteins are known to have a relationship with themselves, e.g. homodimerization, represented in Fig. 1a as a dotted line. In the second scenario, shown in Fig. 1b, there is a direct relationship between a drug target and a disease protein. In the third scenario a drug target and disease protein do not have a direct relationship, but there is an indirect relationship between them that goes through an intermediate protein (Fig. 1c). To keep the graph comprehensible, we made the choice to use paths with a maximum length of two, i.e. paths with at most one intermediate protein, for indirect relationships. Guney et al. previously showed that these paths cover 90% of the relationships between drug targets and disease proteins, and that using longer paths does not improve performance [13].

### Reference sets

To evaluate and benchmark the performance of our method, we used the reference set created by Guney et al. [13]. Furthermore, we created a second reference set using a similar procedure as Guney et al. However, whereas Guney combined related diseases into disease classes that minimally have 20 disease proteins, the second reference set includes any disease for which disease proteins are known.

#### The Guney reference set

The reference set created by Guney et al. [13] consists of 402 “known” drug-disease combinations. Table 1 provides an overview of the characteristics of this reference set. The reference set contains 238 drugs, each of which is represented by a unique set of drug targets. The 78 diseases contained in the reference set are represented by an equal number of disease protein sets, of which 74 sets are unique.

**Table 1:** Characteristics of the two reference sets

Characteristics	Guney reference set	EMC reference set
Source of drug-disease indications	MEDI-HPS + Metab2MeSH + manual curation	MEDI-HPS [23]
Drug target sets	238	314
Unique drug targets	384	539
Source of drug targets	DrugBank	Santos et al. [25]
Disease protein sets	78	281
Unique disease proteins	2726	3205
Minimum size of disease protein set	20	1
Median size of disease protein set	52	5
Maximum size of disease protein set	606	273
Source of disease proteins	OMIM + GWAS	DisGeNet, curated subset [26]
Number of positive cases	402	1250
Number of negative cases	18,162	86,984

Disease proteins were obtained from the Online Mendelian Inheritance in Man (OMIM) database and GWAS studies, and drug targets were obtained from DrugBank. The drug indications were obtained from the Medication Indication – High Precision Subset (MEDI-HPS) [23], which was further filtered for strong literature evidence by using the Metab2MeSH tool [24]. Finally, Guney et al. manually checked all drug labels to confirm that they were used to treat the disease. A complimentary set of “unknown” combinations was created by taking all possible drug-disease combinations, excluding the 402 “known” combinations. This “unknown” set consisted of drug-disease combinations that are likely to be ineffective. In the following, we shall refer to the “known” and “unknown” combinations as positive and negative combinations, respectively, to align with common terminology in the machine learning field.

The Guney reference set only included a disease if at least 20 disease proteins were associated with it. This criterion biases the reference set towards well-characterized diseases. Furthermore, diseases with fewer disease proteins were rolled-up based on the MeSH hierarchy until a MeSH entry with 20 or more disease proteins was found. As a result, the reference set contains entries such as “neoplasms”, “bone diseases”, “kidney diseases”, and “autoimmune diseases”, which are better described as disease classes rather than individual diseases.

#### The EMC reference set

The second reference set, which we refer to as the EMC (Erasmus Medical Centre) reference set, covers 708 drugs, represented by 314 unique sets of drug targets. Its 285 diseases are represented by 281 unique sets of disease proteins, the minimum set size of which is lowered to 1. The resulting reference set consists of 1250 unique combinations of drug target and disease protein sets (Table 1).

Drug targets were obtained from the review by Santos et al. [25], which is stated to describe a more comprehensive and consistent list of drug targets than DrugBank. The disease proteins were obtained from DisGeNet, from which we used the manually curated subset [26]. Drug indications were obtained from MEDI, which extracts them from the literature with an ensemble text-mining pipeline [23]. We used the “high precision” subset (HPS) of MEDI, as provided by the authors. Similar to the Guney reference set, negative cases were created by taking all possible combinations of the sets of drug targets and disease proteins, excluding the “known” combinations.

### Feature sets and machine learning

**Fig. 2:**
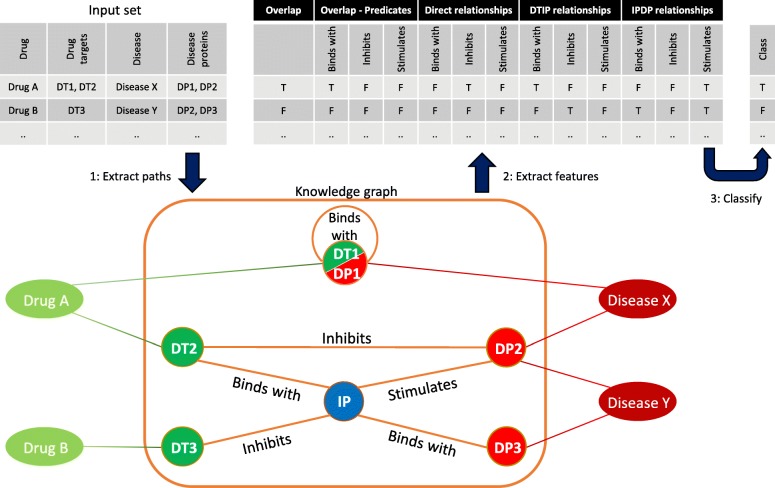
Schematic overview of the feature extraction and classification process. For the sake of readability, this overview figure only shows the process for predicates. The input set contains the combinations of drug targets (DT) and disease proteins (DP) that are to be classified. Step 1: Extract paths. The paths between drug targets and disease proteins are extracted from the knowledge graph. Paths can be direct or indirect. Indirect paths have one intermediate protein (IP) and are separated in two steps: DTIP (drug target – intermediate protein) and IPDP (intermediate protein – disease protein). Step 2: Extract features. The feature set consists of all possible predicates and provenance, for each of the three scenarios (cf. Fig. 1). Based on the extracted paths for a combination, the presence or absence of each feature is set. Step 3: Classify. Based on the extracted features, the combinations are classified by a random forest classifier

We used the three scenarios of drug target-disease protein combinations (see Fig. 1) as the basis for our feature generation. For each scenario, a binary feature table was created of all predicates and corresponding provenance (Fig. 2):

1. Overlap, a binary feature which indicated whether one or more disease proteins were also drug targets. If a protein had a relationship with itself in the knowledge graph (for example because it homodimerizes), this information was also included as binary features. However, such self-relationships were not available for all overlapping proteins.
2. Direct relationships, filling in the binary table of the predicates and provenance of all the direct relationships between drug targets and disease proteins.
3. Indirect relationships, split into two steps as shown in Fig. 1c. For each step, a binary table of the predicates and provenance was filled in.

To assess and quantify the value of predicate and provenance information, we performed multiple experiments with variations of the feature sets:

- A baseline was set by classifying drug target-disease protein combinations without any predicate and provenance information. To do so, three binary co-occurrence features were created: “overlap”, “has_direct_relationship”, and “has_indirect_relationship”. These features indicate whether the paths between the drug targets and disease proteins belong to one or more of the three scenarios described earlier.
- Drug target-disease protein combinations were classified using only the predicate features or only the provenance features, thereby quantifying the value of each.
- Drug target-disease protein combinations were classified with both predicate and provenance information, which constituted the complete feature set.
- All the features from the overlapping drug targets and disease proteins and the direct relationships were removed. Removing these features allowed us to quantify the dependence of our method on the proximity between drug targets and disease proteins.

Random forests were trained to classify a combination of drug targets and disease proteins as positive or negative. We chose random forests because they have shown excellent performance as compared to other classifiers on a wide range of problems [27], and they can calculate the importance of individual features. Classifications were performed on all positive combinations and an equally-sized sample of randomly selected negative combinations. Classification performance was assessed by the area under the ROC curve (AUC) of a 10-fold cross-validation experiment [28]. We report the mean and standard deviation of the AUCs of 100 repeated cross-validation experiments. For the classifications that were performed on the complete feature sets we also report the area under the precision and recall curve (AUPR) [29].

To compare our method with the proximity-based method of Guney et al., we implemented their method with the code provided on GitHub [30]. As an input, we used the protein-protein interaction data extracted from the EKP.

Feature extraction, machine learning, and evaluation were performed in R [31] with the packages caret [32], randomForest [33], pROC [34], and PRROC [35].

## Results

### Extracted paths

We extracted 1.58 million triples with proteins both as subject and object from the EKP, involving a total of 15,124 proteins. Almost a third of these, 4899, were disease proteins for one or more diseases. From a total number of 706 drug target proteins in the two reference sets, there were 425 (60%) which were also disease proteins. Drug targets and disease proteins were connected by 267,032 direct paths, and almost 50 million indirect (two-step) paths. In total, there were 1.45 million edges between the proteins. The triples were taken from 25 different knowledge sources [see Additional file 1: Table S1], and contained 45 different predicate types [Additional file 1: Table S2]. A small percentage of the disease proteins (3% for the Guney reference set and 1% for the EMC reference set) were not used in the analyses because the EKP did not contain a direct or two-step indirect path between the disease protein and a drug target. All drug targets were connected to a disease protein through a direct or indirect path.

### Classification results

The outcomes of all experiments are shown in Table 2, which lists the mean AUC values and their standard deviations for both reference sets. The baseline performance of our reduced feature set, consisting of overlap and co-occurrence features, was 59.8% for the Guney reference set and 64.9% for the EMC reference set. Both predicate features and provenance features substantially improved performance as compared to baseline. Using only predicate features achieved a higher performance than provenance features, performing almost equal to classifying with the complete feature sets. The combination of predicate and provenance features performed best, with an AUC of 78.1% for the Guney reference set, and 74.3% for the EMC reference set. The mean and standard deviation of the AUPR for these two sets was 80.3% (1.5%) and 76.5% (0.9%), respectively. When only features from indirect relationships were used, the mean AUC for both reference sets decreased by 3.7 percentage points.

**Table 2:** Performance results for different feature sets

Feature set	AUC Guney reference set	AUC EMC reference set
Overlap and co-occurrence features	59.8% (0.9%)*	64.9% (0.6%)
Overlap and predicate features	77.6% (1.6%)	73.1% (0.9%)
Overlap and provenance features	75.1% (1.7%)	71.3% (1.0%)
Overlap, predicate and provenance features (all relationships)	78.1% (1.7%)	74.3% (1.0%)
Predicate and provenance features (indirect relationships only)	74.4% (1.9%)	70.6% (1.0%)
Guney’s proximity metric	65.6% (1.4%)	64.6% (0.6%)

*Values indicate mean and standard deviation of the AUCs of 100 experiments

Application of Guney’s proximity metric to the paths extracted from the EKP resulted in an AUC of 65.6% for the Guney reference set, similar to the AUC of 66% that was previously reported [13]. Classification of the EMC reference set based on their proximity metric achieved an AUC of 64.6%. Comparing our method with Guney’s method, we see an improvement in AUC of 12.5 percentage points for the Guney reference set, and of 9.7 percentage points for the EMC reference set.

To ensure a valid comparison with the work of Guney et al., we used a balanced training set with an equal number of positive and negative cases. We assessed the effect of varying the ratio of positive and negative cases in the training set, and found that an increase in the number of negative cases slightly improved performance for the Guney reference set (up to AUC 80.8% for a 10:1 ratio) [see Additional file 2]. For the EMC reference set, no performance improvement was found.

### Importance of predicates and provenance

**Fig. 3:**
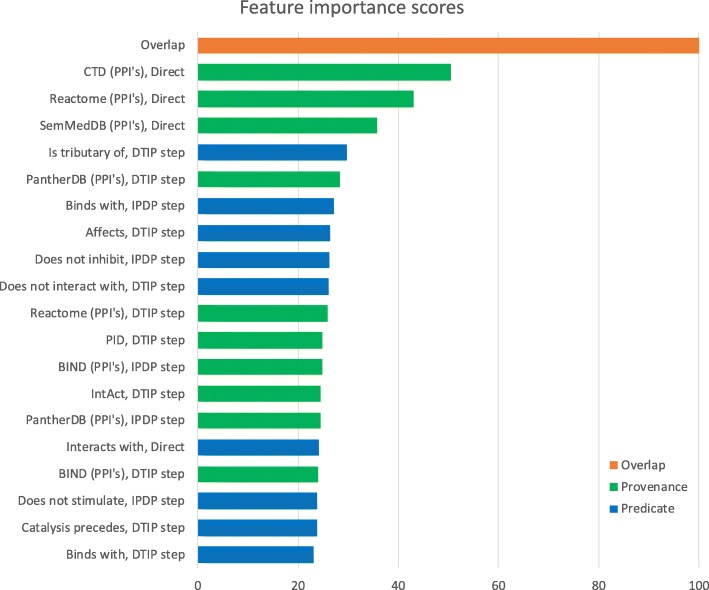
The most important features for a cross-validation experiment. The top-20 most important features when trained on the complete feature set are presented. The importance measures, calculated with the standard feature importance calculation function of the random forest algorithm, have been normalized. The colours indicate whether it is a predicate, provenance, or overlap feature. While knowledge sources such as SemMedDB contain information about relationships between many types of entities, we only used the protein-protein interaction (PPI) subsets of these datasets

To determine the importance of individual features, we used the standard feature importance calculation function of the random forest algorithm. Figure 3 shows the ranking of the 20 most important features for one of the cross-validation experiments. This experiment was performed on the EMC reference set, using the full feature set. The overlap feature was most important, followed by three provenance features from direct relationships. The number of predicate features in the top-20 most important features was about the same as the number of provenance features.

We also examined the importance of the individual knowledge sources. For each source in turn, we removed the features that could only be derived from that source from our feature set and performed a 10-times repeated 10-fold cross validation. For most knowledge sources, exclusion barely changed the AUC. For only two knowledge sources, exclusion resulted in a decrease in AUC of more than one percentage point. These were the protein-protein interaction subsets from SemMedDB, whose exclusion caused a decrease of 1.2 percentage point, and the Comparative Toxicogenomics Database (CTD), whose exclusion caused a decrease of 2.8 percentage points.

### Influence of number of drug targets on classification performance

The number of drug target proteins per drug varies considerably, ranging from 1 to 26 for the Guney reference set (mean of 3.5, median of 2), and from 1 to 51 for the EMC reference set (mean of 3.4, median of 2). To investigate whether classification performance was dependent on the number of proteins targeted by a drug, we stratified both reference sets into three subsets: combinations with one drug target, with two drug targets, and with more than two drug targets. We then calculated the performance of each subset based on the cross-validation results of all combinations. The performance of the individual subsets within these experiments is shown in Table 3. Performance decreased when only a single drug target was known, while performance increased for drugs with more than one drug target.

**Table 3:** Classification performance stratified by the number of proteins targeted by a drug

Number of targets per drug	Guney reference set		EMC reference set	
	Number of combinations	AUC	Number of combinations	AUC
1	133	71.8% (2.9%)*	552	71.8% (1.4%)
2	125	78.5% (2.4%)	244	75.6% (1.5%)
> 2	144	82.4% (2.2%)	454	76.6% (1.5%)
All	402	78.1% (1.7%)	1250	74.3% (1.0%)

*Values indicate the mean and standard deviation of the AUCs for 100 experiments

### Error analysis

We analysed the errors of one cross-validation experiment on the EMC reference set, which achieved an AUC of 73.8%. The random forest classifier assigned probabilities to the combinations. In the following, we qualitatively analyse misclassifications from the extremes of the distribution of these probabilities.

#### Qualitative analysis of false negatives

We further examined the eight false-negative cases with a probability of less than 0.1. Four cases were classified as negative because the diseases involved had only a single associated disease protein, and the EKP did not contain direct or two-step indirect paths between the disease protein and the drug targets.

We considered two of the four remaining cases to be clear classification errors. According to the product label of colchicine, it is a valid treatment for familial mediterranean fever [36]. Similarly, treating gastric immobility with the gastrokinetic agents tegaresod and cisapride is described in the literature [37].

The other two false-negative classifications were less straightforward. One of these described the use of steroids such as prednisolone or hydrocortisone to treat otitis media. While this appears to be a commonly accepted treatment [38], its validity has recently been disputed [39]. Treating kidney stones with diuretics such as hydrochlorothiazide and polythiazide functions by increasing renal clearance, thereby excreting any substance aggregates before they become kidney stones [40]. This treatment does not influence the mechanisms that cause the substance to form, but instead prevents their build-up from reaching problematic concentrations.

#### Qualitative analysis of false positives

We similarly investigated the 15 false-positive cases with random forest probabilities larger than 0.9. Two cases appeared to be incorrectly marked as negative in our reference set. Low levels of brain natriuretic peptide are known to be associated with hypertension [41], and amphetamines such as dextroamphetamine and lisdexamfetamine are an accepted treatment for narcolepsy [42, 43].

For six cases, efficacy has been investigated, but the drug proved to be ineffective or no conclusion about its efficacy could be drawn. For example, the vasopressin receptor antagonist tolvaptan appeared to be effective against the X-linked subclass of nephrogenic diabetes insipidus [44]. However, since this finding was published in 2006, the drug does not appear to have become an accepted treatment [45]. Dextromethorphan has more recently been described as a potential treatment for bipolar disorder, and is currently under investigation in a clinical trial [46, 47]. However, at the time of writing no results have been published. The 5HT-antagonist ondansetron was tested as a treatment for Alzheimer’s disease, but failed to improve cognitive performance [48].

Five other drugs appeared to be potential causes rather than treatments of the disease. For example, the drug label of sotalol mentions that it can cause asthma [49], and the drug label of pentoxifylline mentions that it can cause hypotension [50].

For two false-positive cases we could not find any relationship in the literature: the use of carboprost to treat acidosis, and of thrombin inhibitors to treat rheumatoid arthritis do not appear to have been investigated. While some animal studies mention the use of thrombin inhibitors for research on rheumatoid arthritis, the use of these drugs appears to be part of a laboratory procedure, not an experimental treatment [51, 52].

## Discussion

We used a biomedical knowledge graph to extract features for the automated classification of efficacious relationships between drug targets and disease proteins. We have shown that the use of predicate and provenance information that is available in the knowledge graph substantially improves classification performance as compared to not using this information. To our knowledge we are the first to use provenance information in a computational analysis. We performed our analysis on an existing, commercially available knowledge graph, saving us the considerable amount of time and effort required to integrate the knowledge sources with each other.

Compared to our baseline performance, which was only based on co-occurrence information, using either predicate or provenance information substantially improved the classification results. With both reference sets, using only predicate information achieved a higher performance than only using provenance information, while using both performed best. In all experiments, use of predicate and provenance information surpassed the performance of the method against which we benchmarked, the state-of-the-art work by Guney et al. [13]. Performance improved for drugs with more than one drug target, or when 20 or more disease proteins were known. Removal of the overlap and direct relationship features, which included the four most important ones, showed that our method can still be used when only indirect paths are available. A lack of proximity between drug targets and disease proteins can therefore be compensated with predicate and provenance information. Excluding the information from a single knowledge source generally had a minor impact on the performance of our method. The largest performance decrease (2.8 percentage points in AUC) was noted for the exclusion of the protein-protein interactions from CTD. Using more negatives cases than positive cases in the training set may slightly increase classification performance.

We created the EMC reference set to analyse diseases with less than 20 disease proteins. This both increased the number of diseases that could be included, and allowed its disease entries to be more specific than the disease classes included by Guney et al. However, our error analysis revealed that the EMC reference set was not perfect, with one of its positive cases likely to be outdated, while in another positive case the drug mitigated the symptoms of the disease rather than treating its underlying causes. The negative cases in the reference set were created by randomly combining the positive cases. While this is a common approach [13, 15, 53], it assumes that there are no undiscovered or missing relationships. Our error analysis showed this assumption to be incorrect, with at least two of the negative cases having a therapeutic relationship in reality. Overall, our work would benefit from a comprehensive gold standard, which would ideally consist of positive and negative cases that have been manually verified by experts.

Expert knowledge could also be leveraged to prune the proteins, predicates, and provenance found in the paths. In this research, the paths that we extracted from the knowledge graph were immediately used to create the features. It is therefore possible that some erroneous paths were included in the analyses.

The feature set could be expanded with other types of information. Network topology features could be used, e.g. the centrality of drug targets and disease proteins in the graph, as previously used by Mitsopoulos et al. [54] and Xu and Li [55], or the proximity metric of Guney et al. [13]. Furthermore, other types of entities, such as physiological or molecular processes could be added to the paths. Finally, more detailed analyses of the provenance underlying triples could be used to create features. In cases where references to journal articles are available in the triples, these can be used to obtain the journal name, author information, and publication date. The value of this information has already been demonstrated by Heinemann et al. [56], who used temporal publication patterns of articles, as well as the number of times a single author published about a drug target to predict the failure of drugs in phase II/III trials.

Finally, we could apply our method to other tasks. Combinations of drugs, e.g. those in the Drug Combination Database [57], could be analysed by combining sets of drug targets. Similarly, comorbidity [58] or diseases trajectories [59] could be analysed by combining sets of disease proteins. Furthermore, we would like to investigate whether our method can be used to identify drugs or drug targets for rare diseases. Because rare diseases receive less attention from the scientific community, their relevant proteins may be more poorly characterized. In such cases our cut-off of two steps might be insufficient, as it already was for four (0.3%) of the positive cases in the EMC reference set, which would necessitate adding another step to the extracted paths. Our method may also be suitable for predicting side effects of drugs, which was a common error in our analysis of the false-positive cases. For this task, the relationships of drug targets with proteins that induce side effects would be analysed, instead of their relationships with disease proteins [13].

## Conclusions

We have demonstrated the added value of predicate and provenance information for knowledge-graph analyses. By achieving a state-of-the-art performance for drug efficacy screening, our work contributes to the computational analysis of the comprehensive body of biomedical knowledge.

## Additional files

Additional file 1: Description of the predicates and provenance which were extracted from the knowledge graph. This file contains two tables. Table S1 describes the number of triples contributed by the knowledge sources and number of unique proteins connected by the triples. Table S2 shows the 45 predicates that connect proteins in the knowledge graph and were used as features. (DOCX 24 kb)

Additional file 2: Performance for different ratios between the positive and the negative cases in the training set. This file shows the performance on a balanced test set as a function of the ratio of positive and negative cases in the training set. (XLSX 14 kb)
