# Supplementary material for: Using predicate and provenance information from a knowledge graph for drug efficacy screening
Source: J Biomed Semantics. 2018 Sep 6;9:23. doi: 10.1186/s13326-018-0189-6 (PMC6127943; doi:10.1186/s13326-018-0189-6)
Supplement: Supplementary file 1 — Description of the predicates and provenance which were extracted from the knowledge graph. This file contains two tables. Table S1 describes the number of triples contributed by the knowledge sources and number of unique proteins connected by the triples. Table S2 shows the 45 predicates that connect proteins in the knowledge graph and were used as features. (DOCX 24 kb) [file 13326_2018_189_MOESM1_ESM.docx]

# Additional file 1

Table S1 Number of triples contributed by the knowledge sources and number of unique proteins connected by the triples.

| Knowledge source | Number of triples | Number of proteins |
| --- | --- | --- |
| IntAct | 1,081,605 | 10,411 |
| SemMedDB | 106,573 | 9418 |
| BioGRID | 102,321 | 11,495 |
| Reactome | 97,948 | 6003 |
| TRANSFAC | 79,776 | 10,306 |
| Human Protein Reference Database | 47,252 | 8114 |
| CTD | 36,544 | 10,565 |
| PID | 27,542 | 2678 |
| Panther database | 23,377 | 1987 |
| Recon X | 21,545 | 1415 |
| CORUM | 18,421 | 2447 |
| KEGG | 14,864 | 1306 |
| BIND | 12,218 | 5407 |
| HumanCyc | 11,370 | 1771 |
| PhosphoSite | 8923 | 1864 |
| DIP | 5100 | 2358 |
| Entrez Gene | 337 | 508 |
| Drugbank | 185 | 119 |
| OMIM | 175 | 114 |
| MTH | 53 | 97 |
| YeastCyc | 37 | 9 |
| NCI | 3 | 6 |
| Cross Species Phenotype Ontology | 8 | 7 |
| MeSH | 2 | 4 |
| PDQ | 2 | 3 |

Table S2 List of the 45 predicates that connect proteins in the knowledge graph and were used as features.

| Affects | Is a |
| --- | --- |
| Augments | Is associated with |
| Binds with | Is compared with |
| Catalysis precedes | Is functionally related to |
| Coexists with | Is higher than |
| Consumption controlled by | Is lower than |
| Controls expression of | Is manifestation of |
| Controls phosphorylation of | Is not higher than |
| Controls state change of | Is not lower than |
| Controls transport of | Is not part of |
| Converts to | Is parent of |
| Disrupts | Is part of |
| Does not coexist with | Is spatially related to |
| Does not convert to | Is the same as |
| Does not inhibit | Is tributary of |
| Does not interact with | Is variant of |
| Does not produce | Manages |
| Does not stimulate | Ortholog is associated with |
| Forms protein complex with | Performs |
| Gene product is biomarker type | Produces |
| Gene product variant causes | Stimulates |
| Inhibits | Uses |
| Interacts with |  |
